# A Woman With Abdominal Pain

**DOI:** 10.1016/j.acepjo.2025.100267

**Published:** 2025-10-29

**Authors:** Fu Chi, Cheng-Han Chen

**Affiliations:** 1 Emergency Department, Taipei Veterans General Hospital, Taipei, Taiwan; 2 School of Medicine, National Yang Ming Chiao Tung University, Taipei, Taiwan

## 1 Patient Presentation

A 67-year-old woman presented to the emergency department with a 2-day history of intermittent chest tightness radiating to the back, accompanied by abdominal pain. On arrival, she was hemodynamically stable (blood pressure, 147/70 mm Hg; heart rate, 95 beats per minute). Abdominal examination was unremarkable. Laboratory evaluation revealed a white blood cell count of 6040/μL and a serum lactate level of 21.9 mg/dL.

**Figure 1:**
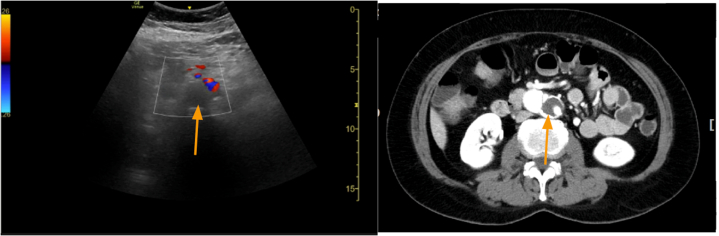
Point-of-care ultrasonography with color Doppler (left) showed a filling defect in the abdominal aorta (arrow), compatible with the findings in computed tomography angiography (right).

Point-of-care ultrasonography with color Doppler demonstrated a filling defect in the abdominal aorta (Video 1). Computed tomographic angiography confirmed a Stanford type B aortic dissection with radiologic malperfusion of the true lumen (Fig 1). She was admitted with a diagnosis of type B aortic dissection complicated by bowel angina, attributed to compression of the true lumen at the level of the abdominal aorta and ostial stenosis of the superior mesenteric artery.

**Video 1:**
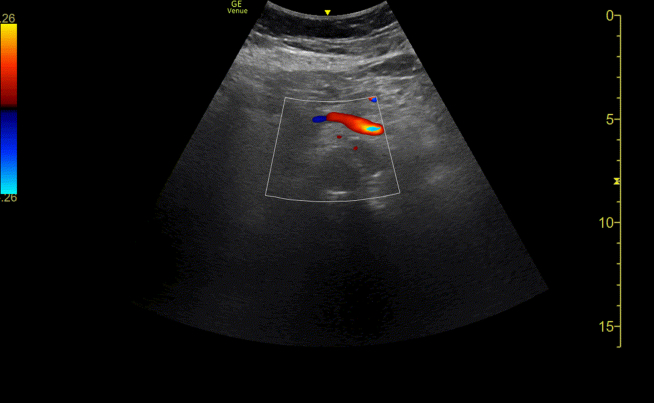
Point-of-care ultrasonography with color Doppler shows a filling defect in the abdominal aorta.

Type B aortic dissection with malperfusion: Type B aortic dissection (TBAD) occurs in 0.5 to 6.3 per 100,000 person-years.^1^ Malperfusion syndrome, present in 20% to 30% of cases, significantly increases morbidity and mortality.^2^^,^^3^ Early recognition is crucial as it transforms uncomplicated TBAD into a surgical emergency requiring immediate intervention.^4^

Point-of-care ultrasonography can provide rapid bedside assessment of aortic pathology. Although computed tomography angiography remains the gold standard, ultrasonography offers real-time evaluation of flow dynamics and can detect filling defects suggesting true lumen compression.^4^ In this case, the elevated lactate (21.9 mg/dL) indicated tissue hypoperfusion,^2^ prompting urgent imaging.

Complicated TBAD with malperfusion requires emergent endovascular or surgical intervention as optimal medical therapy alone is insufficient. Early ultrasonographic detection enables timely recognition of this life-threatening condition, potentially improving outcomes through rapid therapeutic intervention.^2^

## Funding and Support

This work was supported by the National Science and Technology Council, Taiwan (Grant No. NSTC 114-2221-E-075-006), and by Taipei Veterans General Hospital, Taiwan (Grant No. V114B-001).
