# Supplementary figures and images for: A Woman With Abdominal Pain
Source: J Am Coll Emerg Physicians Open. 2025 Oct 29;6(6):100267. doi: 10.1016/j.acepjo.2025.100267 (PMC12641217; doi:10.1016/j.acepjo.2025.100267)

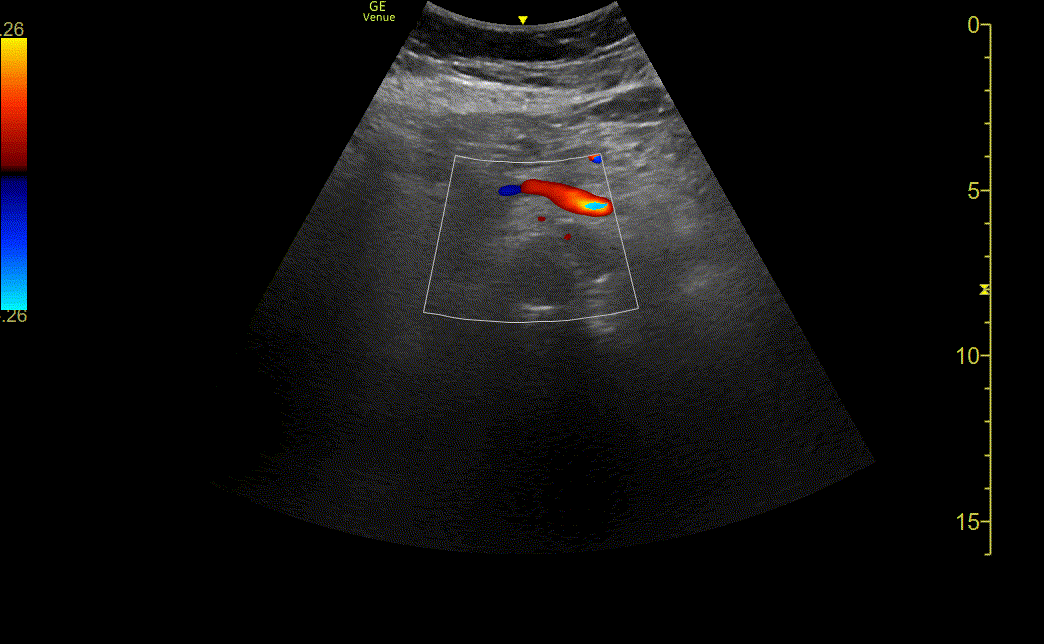

Supplement: Supplementary file 1 [file mmc1.gif]
